# Anti-tumour activity in vitro and in vivo of selective differentiating agents containing hydroxamate

**DOI:** 10.1038/sj.bjc.6690493

**Published:** 1999-06

**Authors:** L Qiu, M J Kelso, C Hansen, M L West, D P Fairlie, P G Parsons

**Affiliations:** Queensland Cancer Fund Laboratories, Queensland Institute of Medical Research, Herston, Queensland, 4029, Australia; Centre for Drug Design and Development, The University of Queensland, Brisbane, Queensland, 4072, Australia

**Keywords:** differentiating agents, melanoma, histone acetylation

## Abstract

A series of hydroxamates, which are not metalloprotease inhibitors, have been found to be selectively toxic to a range of transformed and human tumour cells without killing normal cells (fibroblasts, melanocytes) at the same concentrations. Within 24 h of treatment, drug action is characterized by morphological reversion of tumour cells to a more normal phenotype (dendritic morphology), and rapid and reversible acetylation of histone H4 in both tumour and normal cells. Two hydroxamates inhibited growth of xenografts of human melanoma cells in nude mice; resistance did not develop in vivo or in vitro. A third hydroxamate, trichostatin A, was active in vitro but became inactivated and had no anti-tumour activity in vivo. Development of dendritic morphology was found to be dependent upon phosphatase activity, RNA and protein synthesis. Proliferating hybrid clones of sensitive and resistant cells remained sensitive to ABHA, indicating a dominant-negative mechanism of sensitivity. Histone H4 hyperacetylation suggests that these agents act at the chromatin level. This work may lead to new drugs that are potent, and selective anti-tumour agents with low toxicity to normal cells. © 1999 Cancer Research Campaign


*British Journal of Cancer* (1999) **80**(8), 1252–1258
© 1999 Cancer Research Campaign
Article no. bjoc.1999.0493

### Anti-tumour activity in vitro and in vivo of selective differentiating agents containing hydroxamate

**L Qiu[1], MJ Kelso[2], C Hansen[1], ML West[2], DP Fairlie[2] and PG Parsons[1]**

[1]Queensland Cancer Fund Laboratories, Queensland Institute of Medical Research, Herston, Queensland 4029, Australia; [2]Centre for Drug Design and Development, The University of Queensland, Brisbane, Queensland 4072, Australia

**Summary** A series of hydroxamates, which are not metalloprotease inhibitors, have been found to be selectively toxic to a range of transformed and human tumour cells without killing normal cells (fibroblasts, melanocytes) at the same concentrations. Within 24 h of treatment, drug action is characterized by morphological reversion of tumour cells to a more normal phenotype (dendritic morphology), and rapid and reversible acetylation of histone H4 in both tumour and normal cells. Two hydroxamates inhibited growth of xenografts of human melanoma cells in nude mice; resistance did not develop in vivo or in vitro. A third hydroxamate, trichostatin A, was active in vitro but became inactivated and had no anti-tumour activity in vivo. Development of dendritic morphology was found to be dependent upon phosphatase activity, RNA and protein synthesis. Proliferating hybrid clones of sensitive and resistant cells remained sensitive to ABHA, indicating a dominant-negative mechanism of sensitivity. Histone H4 hyperacetylation suggests that these agents act at the chromatin level. This work may lead to new drugs that are potent, and selective anti-tumour agents with low toxicity to normal cells.

**Keywords:** differentiating agents; melanoma; histone acetylation

Antitumour agents in current use tend to be cytotoxic but non-selective, indiscriminately killing both normal and tumour cells, and resulting in toxic side-effects and development of resistance. An alternative and perhaps more desirable approach is to selectively convert cancer cells to a differentiated, non-proliferating phenotype. This strategy has the potential to be tissue-specific and to avoid the genotoxic side-effects of current anticancer drugs (Marks et al, 1994; Rifkind et al, 1996). A few differentiating agents of relatively low potency have reached clinical trial (Beere and Hickman, 1993), with retinoic acid showing promise for some lymphoid neoplasms. However, the success of this approach has not been fully explored due to the low potency and lack of selectivity for tumour cells of drugs in this class. Expression of many genes in a particular cell type are also affected by these non-selective agents, confounding mechanistic studies of cell arrest.

Hexamethylene bisacetamide (HMBA) is one such differentiating agent that has low potency in various murine and human leukaemic and solid tumour cell lines (Marks et al, 1994; Rifkind et al, 1996). Nevertheless, it has produced remissions in patients with myelodysplastic syndrome and acute myoblastic leukaemia (Andreef et al, 1992), although it suffers from rapid degradation (deacylation) in vivo and side-effects such as acidosis and neurotoxicity (Marks et al, 1994). The lack of tumour selectivity has been noted (Rowinsky et al, 1992) and combination with other agents has been suggested as a way of enhancing its activity. In murine erythroleukaemic cells, HMBA induces arrest in G1, translocation of protein kinase C (PKC) from the cytosol to the membrane, decrease in c-*myb*, c-*myc* and p53 proteins and increase in c-*fos* mRNA (Marks et al, 1994; Rifkind et al, 1996). A transient increase in hypophosphorylated retinoblastoma protein pRB was found 12 h after treatment, followed by enhanced production of the hyperphosphorylated form ppRB, p107 mRNA, and protein during the next 2–3 days (Kiyokawa et al, 1994; Marks et al, 1994). Binding of the key cell cycle-regulating transcription factor E2F to its DNA motif was inhibited by HMBA within 8 h (Richon and Ventaperez, 1996). HMBA also inhibits poly(ADP)ribosylation (Thyberg et al, 1995) and expression of cyclin A (Nakamura et al, 1995), and causes hypomethylation of DNA (Palliti et al, 1993).

*Received 23 November 1998*
*Revised 2 December 1998*
*Accepted 6 January 1999*

*Correspondence to:* PG Parsons

Recently, a number of HMBA-related agents that contain one or more hydroxamate groups have been examined as differentiating agents (Breslow et al, 1991; Schroy et al, 1994; Richon et al, 1996; Parsons et al, 1997). This behaviour is different from that known for other hydroxamates that inhibit matrix metalloproteases and also show promise as anti-tumour agents (Rasmussen and McCann, 1997). Azelaic bishydroxamate (ABHA) is not a metalloprotease inhibitor but induces differentiation in murine erythroleukaemic cell lines, human promyelocytic cells (HL-60), and human colon carcinoma cells (Breslow et al, 1991; Schroy et al, 1994). This compound and certain analogues are up to 100-fold more active than HMBA in vitro and several have been found to inhibit histone deacetylase (Richon et al, 1998) and induce apoptosis (McBain et al, 1997).

Upon comparing the in vitro effects of azelaic acid, HMBA and ABHA, we found that ABHA was selectively toxic for some human tumour cell lines and SV40-transformed melanocytes compared with normal cells (Parsons et al, 1997). After treatment with ABHA, dendritic morphology was the only detected indicator of increased differentiation, markers for the pigmentation pathway being unchanged or down-regulated. ABHA resembled a more potent form of butyrate in some transcriptional activities, raising the possibility that it regulates gene expression via hyperacetylation of histones. Acetylation of the N-terminal lysine side-chains in histone H4 regulates gene expression by blocking access of transcription and other factors to DNA, the level of histone acetylation being determined by histone acetyl-transferases and deacetylases (Wolffe and Pruss, 1996). Butyrate and the potent differentiating agents trichostatin A (TSA) and trapoxin cause hyperacetylation by inhibiting histone deacetylase (Kijima et al, 1993; Yoshida et al, 1995).

We now show that ABHA and a related hydroxamate are active in vivo against xenografts of a drug-resistant human melanoma cell line as well as being active in vitro against most of the human tumour cell lines tested. We also demonstrate that the action of ABHA requires macromolecular synthesis and phosphatase activity, and causes hyperacetylation of histone H4, in sensitive and resistant cells.

### MATERIALS AND METHODS

### Chemistry

*Azelaic-1-hydroxamate-9-anilide (AAHA)*

Azelaic acid (20 g, 106 mmol) was treated with thionyl chloride (25 ml) and the mixture refluxed for 30 min. Solvent was removed in-vacuo and to the residue was added drop-wise a solution containing aniline (10.65 mL, 117 mmol), *N*-methylmorpholine (12.85 ml, 117 mmol) and dichloromethane (100 ml). After addition was complete, the mixture was allowed to stir at room temperature for 20 min before evaporating the dichloromethane in vacuo. The residue was taken up into 200 ml of 5% sodium hydroxide and extracted with 3 × 100 ml aliquots of ethyl acetate. The basic layer was separated, acidified with 6 M hydrochloric acid (HCl) and extracted with 3 × 100 ml aliquots of ethyl acetate. The organic extracts were combined, dried (magnesium sulphate ($MgSO_4$)) and concentrated to yield the anilide (15.6 g, 56%). To a sample of the crude anilide (200 mg, 0.76 mmol) in dry tetrahydrofuran (20 mL) was added *N*-methylmorpholine (0.092 ml, 0.85 mmol) and isobutylchloroformate (0.110 mL, 0.85 mmol). The mixture was stirred for 10 min before a solution containing hydroxylamine hydrochloride (58.2 mg, 0.85 mmol), ethanol (10 ml) and 5% sodium hydroxide (0.94 ml) was added. After a further 10 min, the solvent was removed in-vacuo and the residue taken up into 100 ml of 1 M HCl and extracted with 3 × 30 ml aliquots of dichloromethane. The organic extracts were combined, dried ($MgSO_4$) and concentrated to yield 130 mg of crude material which was purified by reverse phase high performance liquid chromatography (HPLC) to give 15 mg (7.1%) of the desired *bis*-hydroxamic acid: 1H NMR (300 MHz, DMSO-d 6); d 1.29 (6H, m), 1.48 (2H, m), 1.58 (2H, m), 1.94 (2H, t), 2.28 (2H, t), 7.00 (1H, t), 7.26 (2H, t), 7.58 (2H, d), 8.65 (1H, br.s), 9.85 (1H, s), 10.3 (1H, br.s); 13C NMR (75.6 MHz, DMSO-d 6); d 25.27,28.65, 28.70, 28.75, 32.43, 36.58, 119.19, 123.08, 128.04, 139.51, 169.27, 171.41.

### Cell culture

Cell lines (Maynard and Parsons, 1986) were grown in RPMI-1640 culture medium supplemented with 5% heat inactivated fetal calf serum (FCS), at 37°C in a humidified atmosphere containing 5% carbon dioxide air. Human melanocytes were maintained in culture media supplemented with 10% FCS, 10 ng $ml^{-1}$ TPA (12-*O*-tetradecanoylphorbol-13-acetate) and 6 ng ml cholera toxin. All cell lines were tested monthly for *Mycoplasma* using a Hoescht staining technique (Chen, 1977). Cell survival was determined on cells treated in microtitre plates for 24 h and allowed to recover for 5 days before determination of incorporation of $^3$H-thymidine (Maynard and Parsons, 1986), or reduction of MTT (3-(4,5-dimethylthiazal-2-yl)-2,5-diphenyltetrazolium bromide (Mossman, 1983). These methods give similar results to clonal-type assays or counting cell number (Parsons et al, 1991, 1997).

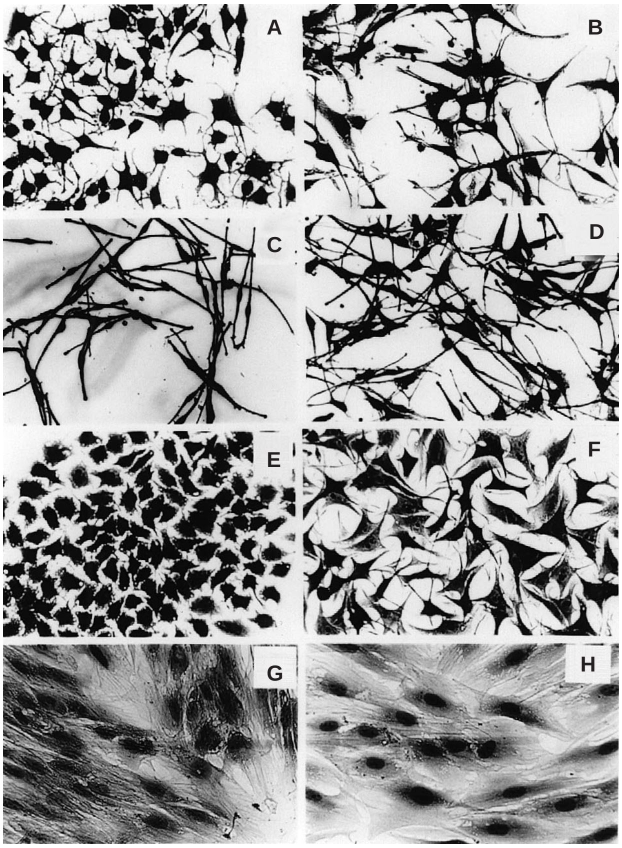

**Figure 1** Effect on cell morphology (Geimsa stain) of 24-h treatment with 100 µg $ml^{-1}$ ABHA. Left panels, untreated. Right panels, treated. (**A,B**) MM96L melanoma; (**C, D**) melanocytes; (**E, F**) HeLa; (**G, H**) NFF fibroblasts

### Cell fusion

The double marker HeLa cell line, HHM, was obtained by selection with 100 µM 2-amino-6-mercaptopurine to generate HPRT-clones, followed by transfection with the plasmid p294MetM3 (Parsons et al, 1997) that expresses hygromycin resistance. HHM cells were resistant to 100 µg $ml^{-1}$ hygromycin B and sensitive to HAT (5 mM hypoxanthine, 20 µM aminopterin, 0.8 mM thymidine).

A2058 or NFF cells were co-cultivated with HHM at a ratio of 10:1 respectively until confluent, in RPMI-1640–10% FCS. The cell monolayer was washed twice with RPMI-1640 serum-free medium and exposed to 500 µl of RPMI-1640 serum-free medium per 50% v/v PEG 1500 for 30 s. Medium was aspirated and cells maintained for a further 60 s. The monolayer was washed stringently (5 ×) with RPMI-1640 serum-free medium and incubated for a further 2 h in 2 ml RPMI-1640 serum-free medium. This was replaced with RPMI-1640 medium supplemented with 10% FCS. Double selection with HAT and 100 µg $ml^{-1}$ hygromycin B was commenced 24 h post-fusion. Colonies were picked and propagated.

*© 1999 Cancer Research Campaign*

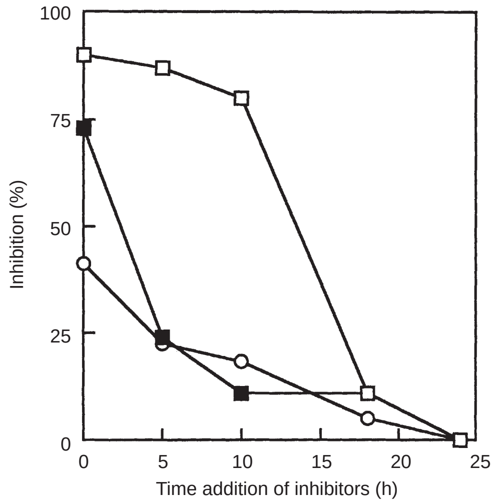

**Figure 2** Formation of dendritic HeLa cells during a 24-h treatment with 100 μg ml$^{-1}$ ABHA was inhibited by co-treatment with 10 μg ml$^{-1}$ cycloheximide (□), 2 μg ml$^{-1}$ actinomycin D (■) or 1 mM sodium vanadate (○). Addition at 0 h represents 24-h treatment with inhibitors, giving maximum inhibition of dendrite formation. Points are means of triplicates

### Mouse experiments

Xenografts of melanoma MM96L were produced by subcutaneous (s.c.) injection of 2 × 10$^6$ cells per site on the flanks of nude (*nu*/*nu*) mice as previously described (Parsons et al, 1991). After 1 week to allow the cells to become established as tumours, the mice were treated once daily (5 days per week) intraperitoneally (i.p.) with 4 mg ABHA in 0.5 ml phosphate-buffered saline (PBS), 5 mg AAHA in 50 μl 80% dimethyl sulphoxide (DMSO) or 25 μg TSA in 0.2 ml PBS. The animals were euthanized when a tumour reached 1 cm diameter.

### Histone acetylation

Histones were extracted in HCl and analysed directly on Triton–urea 15% polyacrylamide gels with Coomassie blue (Saito et al, 1991). Gels were scanned with a laser densitometer (Molecular Dynamics, Sunnyvale, CA, USA) and quantitated with ImageQuant software.

### RESULTS

### Cell morphology and survival

Treatment of normal cells (melanocytes, fibroblasts) with ABHA for 24 h led to some enlargement of the cytoplasm but no major morphological changes (Figure 1). Tumour cells (MM96L, HeLa), became highly dendritic following similar treatment, with the MM96L melanoma cells resembling the morphology of normal melanocytes (Figure 1C, D). Some membrane damage was evident with vesicles forming from the plasma membrane in some cells, as well as cells undergoing apoptosis.

Simultaneous treatment of cells with ABHA and a range of inhibitors demonstrated that formation of dendritic morphology was dependent on protein and RNA synthesis, and to a lesser extent, phosphatase activity (Figure 2).

As found previously in cell growth assays (Parsons et al, 1997), incorporation of $^3$-H-thymidine 5 days after a 24-h treatment confirmed that the human melanoma cell line MM96L and the cervical carcinoma cell line HeLa were sensitive to killing by ABHA compared with fibroblasts (NFF) (Figure 3A). The melanoma cell line MM229 had intermediate sensitivity. AAHA, the anilide derivative of ABHA, showed similar discrimination and was more potent (Figure 3B). TSA (Figure 3C) was approximately 100-fold more potent than the two previous drugs, but was somewhat less selective for killing fibroblasts. A survey of other human cell types (Table 1 and Figure 8A) showed that only two tumour cell lines (MM229 and A2058) of 11 human tumour and transformed cell lines were as resistant to ABHA and AAHA as proliferating normal cells (NFF and NM).

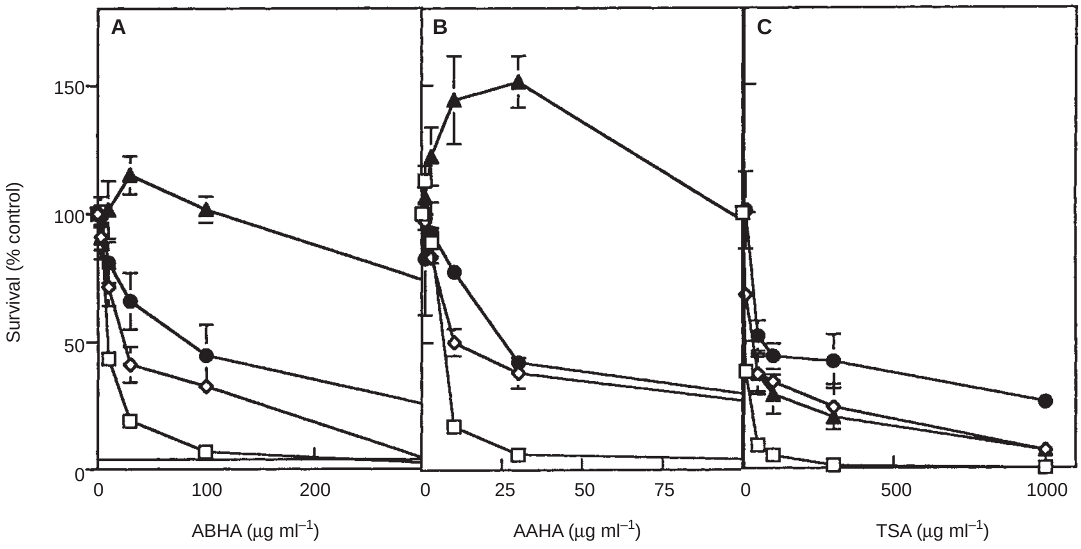

**Figure 3** Selective toxicity of ABHA (**A**), AAHA (**B**) and TSA (**C**) in human cells. ▲, NFF; □, MM96L; •, MM229; ◇, HeLa, Points are mean and s.d. (*n* = 4)

 *© 1999 Cancer Research Campaign*

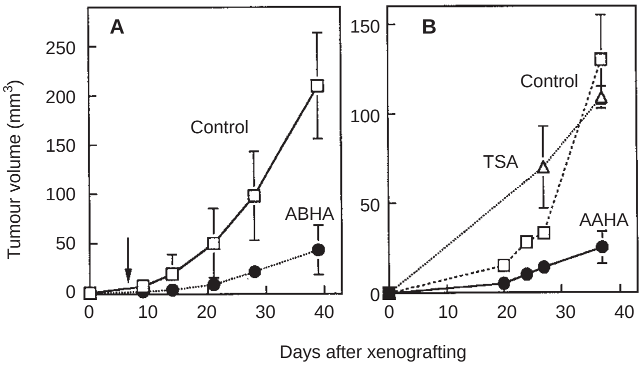

**Figure 4** Inhibition of the growth of the human melanoma cell line MM96L as xenografts in nude mice. (**A**) ABHA (4 mg per day). (**B**) AAHA (5 mg per day) and TSA (25 µg per day). Points are mean and s.d. ($n$ = 7–11 per group)

**Table 1** Toxicity of histone deacetylase inhibitors in human cells

| Cell | Cell type | $D_{37}$[a] | | |
|---|---|---|---|---|
| | | TSA (ng ml$^{-1}$) | ABHA (µg ml$^{-1}$) | AAHA (µg ml$^{-1}$) |
| Normal | | | | |
| NFF | Fibroblasts | 50 | > 300 | > 100 |
| NM | Melanocytes | 170 | 196 | > 100 |
| Tumour | | | | |
| MM96L | Melanoma | 10 | 14 | 8.0 |
| MM229 | Melanoma | 413 | 179 | 26 |
| MM418c1 | Melanoma | 38 | 16 | 6 |
| HT 144 | Melanoma | 120 | 72 | 39 |
| HeLa | Cervical carcinoma | 50 | 56 | 30 |
| A549 | Lung carcinoma | 39 | 28 | 8.7 |
| LIM 1215 | Colon carcinoma | 30 | 26 | 10 |
| C180–13S | Ovarian cancer | 10 | 10 | 8.0 |
| JAM | Ovarian cancer | – | 11 | – |
| Transformed | | | | |
| 293 | Kidney | – | 1 | 86 |

[a]Dose required to lower survival to 37% of control.

### Inhibition of the growth of human melanoma xenografts in nude mice

Preliminary experiments showed that daily i.p. doses of ABHA up to 8 mg had no discernible toxicity to BALB/c mice. ABHA administered at 4 mg per day, commencing 7 days after implantation of tumour cells, led to significant inhibition of tumour growth (Figure 4A). AAHA was also effective, whereas TSA, applied daily at a dose calculated from cell survival curves to be approximately fivefold the in vitro toxicity of ABHA, was inactive (Figure 4B). Incubation of the drugs with near-confluent cultures of NFF or MM96L cells for up to 3 days, followed by bioassay of the remaining toxicity by transfer of the medium to fresh cultures of MM96L cells, revealed that TSA was slowly inactivated (Figure 5).

No weight loss or other evidence of toxicity was observed in the treated mice. The small tumours remaining on the treated mice were re-cultured and found to retain the same in vitro sensitivity to ABHA as MM96L cells from the untreated tumours.

**Table 2** Acetylation of histone H4 during 24-h treatment with differentiating agents

| Cell | Tetra-acetyl-H4 (% total H4)[a] | | |
|---|---|---|---|
| | 0 | ABHA (100 µg ml$^{-1}$) | Butyrate (5 mM) |
| MM96L | 0 | 28 ± 4.3[b] | 18 |
| MM229 | 0 | 23 | 13 |
| HeLa | 0 | 32 | 12 |
| NFF | 0 | 20 ± 1.6[b] | 22 |
| Melanocyte | 0 | 13 | NT |

[a]Histones separated on Triton–urea gels were stained with Coomassie blue and quantitated with a laser densitometer. [b]Mean and s.d. of 2–3 experiments.

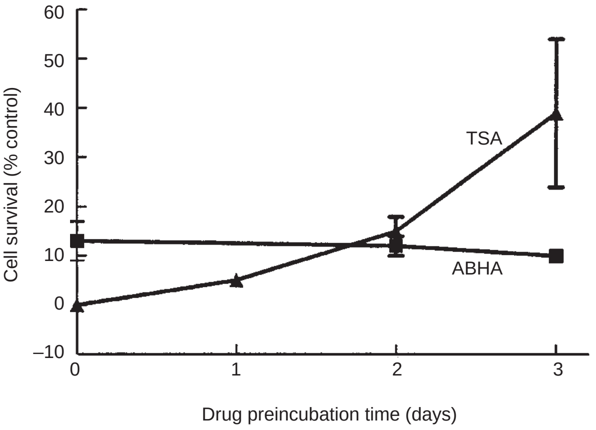

**Figure 5** Loss of potency of TSA during incubation with confluent MM96L cells. At each time, aliquots of culture medium were bioassayed for toxicity by 24-h treatment of separate cultures of MM96L cells. ▲, TSA (100 ng ml$^{-1}$); ■, ABHA (100 µg ml$^{-1}$). Points are mean and s.d. ($n$ = 4)

### Hyperacetylation of histones

Analysis of histones extracted from MM96L or NFF cells after 24 h treatment with ABHA showed a distinct laddering of the H4 region (Figure 6), indicative of increasing levels of acetylation of this histone. Similar results were obtained using 10 µg ml$^{-1}$ AAHA, 100 nM TSA or 5 mM butyrate (not shown). The temporal response, measured from laser densitometer traces (Figure 7A), showed that acetylation commenced within 2 h of treatment, the tetra-acetylated form becoming prominent by 24 h. In repeated experiments, however, no significant difference could be found between MM96L and NFF in the amount of tetra-acetylated form induced by ABHA (Table 2).

Since it was possible that ABHA selectivity for cell killing may have arisen from decreased rate of removal of acetyl groups from H4 histone after removal of drug, cells were analysed at different times during recovery from a 2-h treatment with ABHA. The results, however, revealed that H4 acetylation in the drug-resistant NFF cells decayed more slowly than in the sensitive MM96L cells (Figure 7B), thus ruling out delayed recovery from acetylation as the cause of sensitivity.

*© 1999 Cancer Research Campaign*

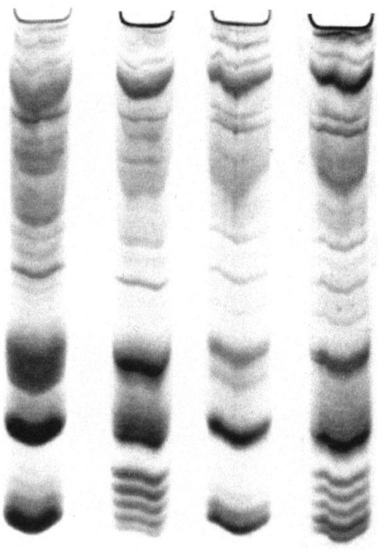

**Figure 6** Separation of histones on a Triton–urea polyacrylamide gel showing induction of histone H4 acetylation in cells treated for 24 h with 100 µg ml$^{-1}$ of ABHA (+)

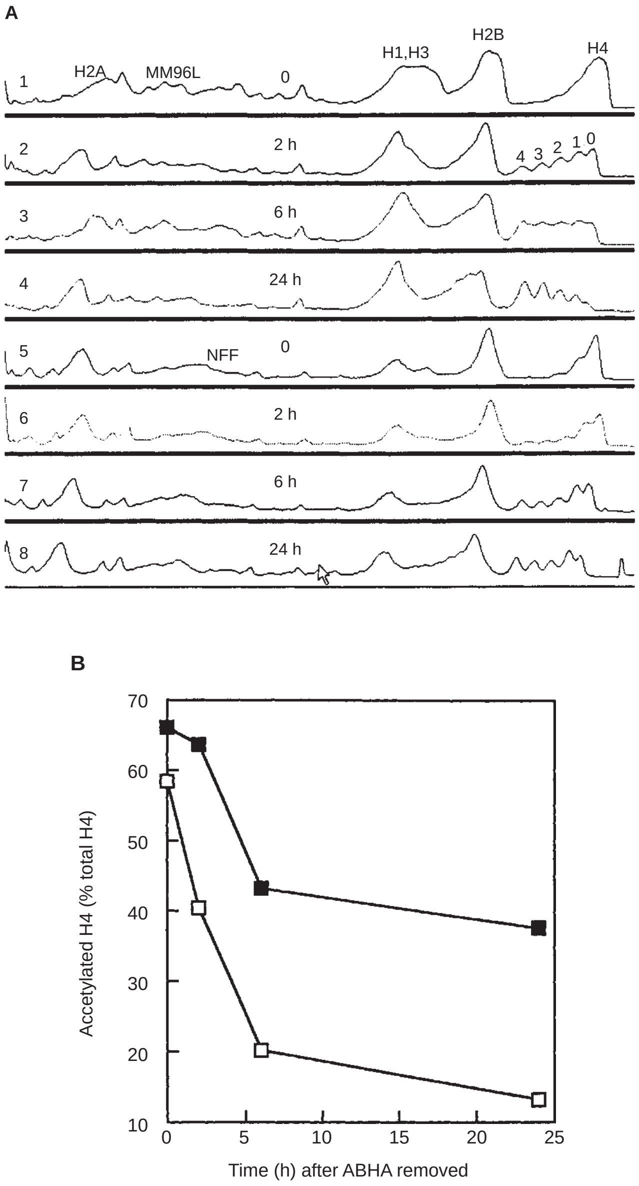

**Figure 7** (**A**) Densitometer profiles of histone gels showing the temporal increase in H4 acetylation in sensitive (MM96L) and resistant (NFF) cells. (**B**) Temporal response for loss of acetylated H4 after a 2-h treatment with 10 µg ml$^{-1}$ ABHA. ■, NFF; □, MM96L

### Sensitivity of cell hybrids to ABHA

Fusion of ABHA-sensitive (HHM) with resistant cells (NFF, A2058) followed by double selection with HAT and hygromycin to remove homokaryons produced stable, proliferating clones. These were identified as hybrids by DNA flow cytometry, which revealed the G1 DNA content to be the sum of the G1 DNA of the fusion partners. The presence of the NFF or A2058 genomes was confirmed independently by increased resistance of the hybrids to the methylating agent *N*-methyl-*N*′-nitro-*N*-nitrosoguanidine compared with HeLa, which is sensitive to DNA methylating agents (Maynard and Parsons, 1986). No clones were obtained from fusions carried out with HeLa cells that lacked either of the selection markers. Clones of both NFF/HHM and A2058/HHM cell hybrids displayed distinct sensitivity to ABHA and AAHA, compared with the resistant partner cell line (Figure 8).

### DISCUSSION

A large number of hydroxamate-containing compounds are now known to possess anti-tumour properties. Most such compounds non-selectively inhibit matrix metalloproteases, which are associated with growth and spread of malignant tumours in models of metastasis, angiogenesis and tumour progression. In contrast, ABHA does not inhibit metalloproteases even at mM concentrations (unpublished), yet has anti-tumour activity. A wide variety of tumour cell types and SV40-transformed melanocytes (Parsons et al, 1997) are sensitive to ABHA and derivatives (Table 1). Selectivity appears to be a novel property of this drug class, since normal cells and only two melanoma cell lines (MM229, A2058) were resistant to the agents.

The morphological changes illustrated in Figure 1 occur within 24 h of treatment with ABHA and are attributed to some form of differentiation or ‘redifferentiation’ and partial reversion to a normal phenotype. While some of the melanoma cells have enhanced dendritic, melanocytic morphology after treatment, there is also some accompanying apoptosis, and the tumour cells ultimately die. Protein and RNA synthesis de novo was required for this phenotype reversion, as found previously for TSA-induced differentiation of HeLa cells (Hoshikawa et al, 1994), suggesting that ABHA is not acting solely as an inhibitor of cellular functions but also induces the transcription of genes necessary for differentiation and possibly cell death.

Xenografts of the melanoma MM96L cells were significantly inhibited by daily treatments with ABHA, the first drug of any kind to show significant activity against this cell line. Although

 *© 1999 Cancer Research Campaign*

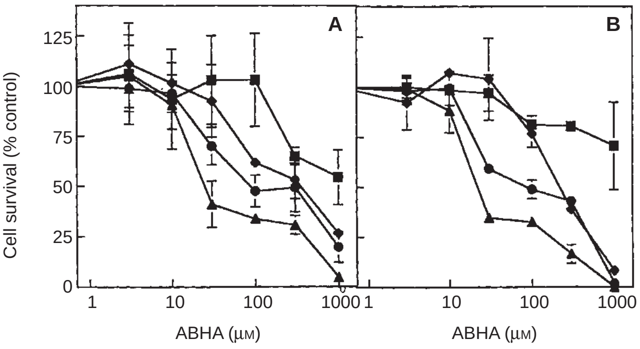

**Figure 8** Dose–response for survival of hybrids between the HeLa fusion partner HHM and A2058 (**A**) or NFF (**B**). ◆, HHM; ■, A2058 (**A**) or NFF (**B**); ● and ▲, hybrid clones. Points are mean and s.d. ($n = 4$)

this is a relatively high dose (200 mg $kg^{-1}$), other differentiating agents such as butyrate have been used safely at similar levels to treat children with sickle cell anaemia (Perrine et al, 1994). Established from a metastasis, having a mutator phenotype (Must et al, 1996) and resistant to current anticancer agents in vitro and in vivo (Maynard and Parsons, 1986), MM96L cells represent a rigorous, but probably realistic, human model for testing new agents. MM96L cells cultured from the small tumours that survived treatment of the mice by ABHA were still sensitive to ABHA. The inability of such a tumour cell line to develop resistance to ABHA is notable, and suggests that even higher doses of ABHA could be given, since there were no overt signs of toxicity or side-effects in the mice. AAHA, administered in DMSO because of poor solubility in aqueous PBS, had even better activity. Metabolic inactivation of TSA, detected here for the first time, is expected to be more marked in vivo due to exposure to the liver and kidney, and is a possible explanation for its complete lack of activity against human melanoma xenografts. Resistance of cultured fibroblasts to TSA is unlikely to be due to metabolism of TSA because the exposure was limited to 24 h. Thus, although TSA is now widely established as a potent inhibitor of histone deacetylases in vitro, metabolically stable inhibitors may be required to obtain useful activities in vivo.

Acetylation of histone H4 was found to be induced by ABHA and AAHA, presumably by inhibition of histone deacetylase activity as shown previously for this class (Richon et al, 1998) and for TSA (Wolffe and Pruss, 1996). This could be expected to profoundly alter gene expression and may be a necessary condition for cell toxicity to occur. However, the present comparison of acetylation and deacetylation rates in intact sensitive and resistant cells failed to find any evidence that differences in H4 acetylation could be responsible for differential toxicity. It is possible that other histone modifications may be involved in selectivity, or that other drug targets are important. It should also be noted that some of the changes in gene expression induced by this type of agent, presumably by histone hyperacetylation, may be remote from the origin of toxicity and selectivity. Protein tyrosine phosphatase activity is involved in regulating cell survival and differentiation (Stoker and Dutta, 1998) and was required for ABHA to differentiate HeLa cells. This may provide a focus for identifying signalling pathways relevant to the selective action of ABHA.

The dominant-negative sensitivity to ABHA of somatic cell hybrids contrasts with the more usual complementation towards resistance found with other agents such as cisplatin (Wang et al, 1996), and in this study with a DNA methylating agent. It could be speculated that ABHA sensitivity involves a dominant, mutated sequence vital to the growth of the transformed phenotype. Loss of this gene or further mutation under ABHA selection pressure would lead to non-viable clones. The similar, dominant-negative phenotype of hybrids from the ABHA-resistant A2058 cells suggests that malignant transformation may not always be accompanied by such changes. The results also show that functional complementation would not be an appropriate method for cloning the gene(s) involved in ABHA sensitivity.

Overall, ABHA and AAHA represent a unique class of histone deacetylase inhibitors that is selectively toxic to transformed cells, stable in culture, and unlike the more potent inhibitor TSA, active against human melanoma cells in vivo with no evidence of adverse effects on the animal or development of drug resistance. Further refinement in structure may be needed to increase potency and hence efficacy in vivo.

### ACKNOWLEDGEMENT

This work was supported by the Queensland Cancer Fund.

### REFERENCES

Andreeff M, Stone R, Michaeli J, Young CW, Tong WP, Sogoloff H, Ervin, T, Kufe D, Rifkind RA and Marks PA (1992) Hexamethylene bis acetamide in myelodysplastic syndrome and acute myelogenous leukemia: a phase II clinical trial with a differentiating agent. *Blood* **80**: 2604–2609

Beere HM and Hickman JA (1993) Differentiation: a suitable strategy for cancer chemotherapy? *Anticancer Drug Des* **8**: 299–322

Boulikas T (1994) A compilation and classification of DNA binding sites for protein transcription factors from vertebrates. *Crit Rev Euk Gene Exp* **4**: 117–321

Breslow R, Jursic B, Yan ZF, Friedman E, Leng, L, Ngo L, Rifkind RA and Marks PA (1991) Potent cytodifferentiating agents related to hexamethylenebisacetamide. *Proc Natl Acad Sci USA* **88**: 5542–5546

Chen TR (1977) In situ detection of *Mycoplasma* contamination in cell cultures by fluorescent Hoechst 33258 stain. *Exp Cell Res* **104**: 255–262

Hoshikawa Y, Kwon HJ, Yoshida M, Horinouchi S and Beppu T (1994) Trichostatin A induces morphological changes and gelsolin expression by inhibiting histone deacetylase in human carcinoma cell lines. *Exp Cell Res* **214**: 189–197

Kijima M, Yoshida M, Sugita K, Horinouchi S and Beppu T (1993) Trapoxin is an irreversible inhibitor of mammalian histone deacetylase. *J Biol Chem* **268**: 22429–22435

Kiyokawa H, Richon VM, Rifkind RA and Marks PA (1994) Suppression of cyclin-dependent kinase 4 during induced differentiation of erythroleukemia cells. *Mol Cell Biol* **14**: 7195–7203

Marks PA, Richon VM, Kiyokawa H and Rifkind RA (1994) Inducing differentiation of transformed cells with hybrid polar compounds: a cell-cycle dependent process. *Proc Natl Acad Sci USA* **91**: 10251–10254

Maynard K and Parsons PG (1986) Cross sensitivity to methylating agents, hydroxyurea and methotrexate in human tumour cells of the Mer phenotype. *Cancer Res* **46**: 5009–5013

McBain JA, Eastman A, Nobel CS and Mueller GC (1997) Apoptotic death in adenocarcinoma cell lines induced by butyrate and other histone deacetylase inhibitors. *Biochem Pharmacol* **53**: 1357–1368

Mosmann T (1983) Rapid colorimetric assay for cellular growth and survival: application to proliferation and cytoxicity assays. *J Immunol Methods* **65**: 55–63

Musk P, Clark JM, Thompson D, Dunn IS, Christopherson RI, Szabados E, Rose SE and Parsons PG (1996) Purine deoxynucleoside metabolism in human melanoma cells with a high spontaneous mutation rate. *Mutat Res* **350**: 229–238

Nakamura T, Okuyama S, Okamoto S, Nakajima T, Sekiya S and Oda K (1995) Down-regulation of the cyclin A promoter in differentiating human embryonal carcinoma cells is mediated by depletion of ATF-1 and ATF-2 in the complex at the ATF/CRE site. *Exp Cell Res* **216**: 422–430

© 1999 Cancer Research Campaign

Palitti F, Carotti D, Busiello V, Bendicenti A, Strom R and DiGirolamo M (1993) DNA hypomethylation and differentiation in Friend leukemia cell variants. *Biochim Biophys Acta* **1216**: 50–54

Parsons PG, Favier D, McEwan M, Takahashi H, Jimbow K and Ito S (1991) Action of cysteaminyl phenols on human melanoma cells in vivo and in vitro: 4-S-cysteaminylphenol binds protein disulphide isomerase. *Melanoma Res* **1**: 97–104

Parsons PG, Hansen C, Fairlie DP, West ML, Danoy P, Sturm RA, Dunn IS, Pedley J and Ablett E (1997) Tumor selectivity and transcriptional activation by azelaic bishydroxamic acid in human melanocytic cells. *Biochem Pharmacol* **53**: 1719–1724

Perrine SP, Olivieri NF, Faller DV, Vichinsky EP, Dover GJ and Ginder GD (1994) Butyrate derivatives. New agents for stimulating fetal globin production in the beta-globin disorders. *Am J Ped Hematol Oncol* **16**: 67–71

Rasmussen HS and McCann PP (1997) Matrix metalloproteinase inhibition as a novel anticancer strategy: a review with special focus on batimatstat and maximastat. *Pharmacol Therap* **75**: 69–75

Richon VM and Ventaperez G (1996) Changes in E2F DNA-binding activity during induced erythroid differentiation. *Cell Growth Diff* **7**: 31–42

Richon VM, Webb Y, Merger R, Sheppard T, Jursic B, Ngo L, Civoli F, Breslow R, Rifkind RA and Monks PA (1996) Second generation hybrid polar compounds are potent inducers of transformed cell differentiation. *Proc Natl Acad Sci USA* **93**: 5705–5708

Richon VM, Emiliani S, Verdin E, Webb Y, Breslow R, Rifkind RA and Marks PA (1998) A class of hybrid polar inducers of transformed cell differentiation inhibits histone deacetylases. *Proc Natl Acad Sci USA* **95**: 3003–3007

Rifkind RA, Richon VM and Monks PA (1996) Induced differentiation, the cell cycle and the treatment of cancer. *Pharmacol Therap* **69**: 97–102

Rowinsky EK, Conley BA, Jones RJ, Spivak JL, Auerbach M and Donehower RC (1992) HMBA in myelodysplastic syndrome: effect of five-day exposure to maximal therapeutic concentrations. *Leukemia* **6**: 526–534

Saito S, Crissman HA, Nishijiima M, Kagabu T, Nishiya I and Cram LS (1991) Flow cytometric and biochemical analysis of dose-dependent effects of sodium butyrate on human endometrial adenocarcinoma cells. *Cytometry* **12**: 757–764

Schroy PC, Rustgi AK, Ikonomu E, Liu XP, Polito J, Andry C and O'Keane JC (1994) Growth and intestinal differentiation are independently regulated in HT29 colon cancer cells. *J Cell Physiol* **161**: 111–123

Stoker A and Dutta R (1998) Protein tyrosine phosphatases and neural development. *BioEssays* **20**: 463–472

Thyberg J, Hultgardh-Nilsson A and Kallin B (1995) Inhibitors of ADP-ribosylation suppress phenotypic modulation and proliferation of smooth muscle cells cultured from rat aorta. *Differentiation* **59**: 243–252

Wang X, Hafezparast M and Masters JR (1996) Genetic basis of drug sensitivity in human testis tumour cells. *Int J Cancer* **65**: 426–431

Wolffe AP and Pruss D (1996) Targetting chromatin disruption: transcription regulators that acetylate histones. *Cell* **84**: 817–819

Yoshida M, Horinouchi S and Beppu T (1995) Trichostatin A and trapoxin: novel chemical probes for the role of histone acetylation in chromatin structure and function. *BioEssays* **17**: 423–430

*© 1999 Cancer Research Campaign*
